# Grylloblattodea of Canada

**DOI:** 10.3897/zookeys.819.24289

**Published:** 2019-01-24

**Authors:** Sean D. Schoville

**Affiliations:** 1 University of Wisconsin-Madison, Department of Entomology, 1630 Linden Drive, 637 Russell Labs, Madison, Wisconsin 53706, USA University of Wisconsin-Madison Madison United States of America

**Keywords:** biodiversity assessment, Biota of Canada, Grylloblattodea, ice crawler, Notoptera

## Abstract

The enigmatic insect order Grylloblattodea comprises two described species in Canada, which are limited to the Montane Cordillera and Pacific Maritime ecozones. One of the described species has three Canadian subspecies of uncertain taxonomic ranking, and there are two additional undescribed or unreported species known in close proximity to the Canadian border in western Alberta and British Columbia that likely also occur in Canada. Thus, as much as 50% of the total taxonomic diversity of Grylloblattodea in Canada is still undocumented. Targeted surveys and taxonomic work, as well as studies that describe the ecology and conservation status of Grylloblattodea are important goals for future research.

In June of 1913, on Sulphur Mountain in Banff National Park, Canada, the entomologists Edmund M. Walker and Takatsuna B. Kurata discovered an unusual insect under stones on a talus slope: “...*Kurata called me to see an insect he had found under a stone... I knew at once that this creature was something new – unlike anything ever found before*” (Hutchinson 2014). This discovery would both captivate and puzzle entomologists for the next century, due to the difficulty in placing it within the phylogeny of insects (Rentz 1982). The “*peculiar wingless thysanuriform insect*” was immediately noted for the presence of an ovipositor in adult females and unusual combination of morphological characters, and so it was described in a new family Grylloblattidae as *Grylloblattacampodeiformis*Walker (1914). Walker argued that the grylloblattid’s morphological features were similar to those found in several “orthopteroid” lineages, to such a degree that he named them by combining the Latin word for cricket (*gryllus*) and roach (*blatta*). They are now placed as sister to the African heel walkers, order Mantophasmatodea, itself discovered in 2002 (Klass et al. 2002, 2003). There is strong genetic support for the Grylloblattodea lineage diversifying from other polyneopteran insects around 150 million years ago (Misof et al. 2014). The common name for North American grylloblattids is widely recognized to be “ice crawler” due to their strong affinities to snowfield habitats, while the more common name “rock crawler” has been used for both Asian and North America lineages that as a group prefer habitats with rocky terrain (Schoville 2010).

Since Kevan (1979) reviewed existing knowledge of the Grylloblattodea (as ‘Notoptera’) in Canada, there has been considerable advancement of our understanding of the evolutionary history and distribution of this group (Kamp 1979, Wipfler et al. 2014), although much remains to be learned about the systematics, ecology and conservation status of these insects (Schoville 2014). Several studies have shown the strong impact of glacial climate history on species diversification and range shifts in both North American and Asian taxa (Schoville and Roderick 2010, Schoville et al. 2013). Notably, the global distribution of Grylloblattodea has been expanded to include the Altai and Sayan Mountains in Southern Siberia, where the Asian genus *Grylloblattella*Storozhenko and Oliger (1984) is found. Additionally, the Korean genus *Namkungia*Kim and Lee (2006) has been described. In North America, five new species of *Grylloblatta* have been described from California and Oregon (Schoville 2012, Marshall and Lytle 2015), bringing the total number of species to 33. One species, *G.campodeiformis*, has four recognized subspecies. In Canada, the number of species has not increased since 1979, remaining at two (Table 1). However, it is widely recognized that a large number of species remain undescribed in North America (Jarvis and Whiting 2006, Schoville and Graening 2013), and the three recognized subspecies of *G.campodeiformis* in Canada (Table 1) are of uncertain taxonomic rank.

Collection records now suggest that ice crawlers are widespread in montane habitats of western Alberta and British Columbia (Schoville and Graening 2013; C Copley unpubl. data). These records remain patchy and are based on passive sampling, making assessments of the distribution, abundance and conservation status of populations difficult. One of the most interesting Canadian localities is Mt. St. Paul in Kamloops, British Columbia (BC), where ice crawlers can be found as low as 400m elevation on south-facing slopes during fall and winter (Gregson 1938, Campbell 1949). It remains to be seen how widespread ice crawlers are in Canada at such low elevation. In addition, a few Canadian distributional records remain disputed, i.e. Forbidden Plateau, Vancouver Island, and Grouse Mountain on the mainland (Spencer 1945, Kamp 1979).

**Table 1.** Census of Grylloblattodea in Canada.

Taxon	No. species reported in Kevan (1979)	No. species currently known from Canada^1^	No. BINs^2^ available for Canadian species	Est. no. undescribed or unrecorded^3^ species in Canada	General distribution by ecozone^4^	Information sources
Grylloblattidae	2	2	1	2	Montane Cordillera, Pacific Maritime	Kamp 1979, Kevan 1979, Schoville and Graening 2013; www.gbif.org; S Schoville unpubl. data

^1^One species, *G.campodeiformis*, has three subspecies reported from Canada. ^2^Barcode Index Number, as defined in Ratnasingham and Hebert (2013). ^3^Also, a fourth subspecies of *G.campodeiformis* is expected to occur in Canada. ^4^See Figure 1 in Langor (2019) for a map of ecozones.

A better understanding of how and when to survey for ice crawlers has emerged in recent years (Schoville and Graening 2013), but it is continually improving with biodiversity studies that focus on winter invertebrate ecology. While traditionally viewed as dependent on snow fields or caves with permanent ice, extensive survey work by Kamp (1973) pointed to the close association of ice crawlers with subterranean rocky environments (hypolithion), where cool and humid conditions maintain viable microhabitats. Our knowledge was recently advanced by a study that discovered extensive foraging activity of ice crawlers in infested and dead conifers in mid-elevation Canadian forests (Esch et al. 2018), and a previous study that showed that ice crawlers are often abundant in these forests even when they are managed for timber harvesting (Huggard and Klenner 2003). Suitable habitats occur in boreal forest, alpine talus slopes, lava fields and other cave sites (Schoville and Graening 2013), though ice crawlers are only likely to be surface active following frost events or when snow is on the ground. Ice crawlers are most readily found at night foraging, but can also be found by turning stones or downed wood, or by searching caves where they are active during daylight hours.

To develop an estimate of the expected number of species in Canada, both distributional data and genetic data (Schoville and Graening 2013, S Schoville unpubl. data) were examined. Due to the close proximity of the type locality of *G.campodeiformisoccidentalis*Silvestri (1931) on the northern slope of Mt. Baker in Washington, this subspecies undoubtedly occurs in adjacent parts of BC. An undescribed species is known from Mt. Spokane, Washington (S Schoville unpubl. data) and is likely to extend throughout the Selkirk Mountains into BC (Pritchard and Scholefield 1978). There are at least two additional undescribed species in the North Cascades of Washington, which are likely to extend into BC. Thus, as much as 50% of the total taxonomic diversity of Grylloblattodea in Canada is still undocumented.

One Barcode Index Number (BIN) is available for Canadian ice crawlers, representing the taxon *G.campodeiformiscampodeiformis*. It should be noted that GenBank holds a large number of accessions for the COII mitochondrial locus, which has been used to diagnose species in both North America and Asia.

An important future effort will be to expand our knowledge of ice crawler species ranges in Canada, as well as invest in ecological studies that measure local abundance and assess possible conservation threats. Further efforts to catalogue, determine the distribution, and genetically sample *Grylloblatta* in Canada are likely to provide general insight into insect biodiversity patterns, especially cold-specialized species, due to the limited dispersal capacity, high levels of endemism throughout North America, and strong genetic structure reflecting past environmental change (Jarvis and Whiting 2006, Schoville and Roderick 2010). A particular focus of surveys in the Coast, Cascade, and Columbia mountain ranges are important to developing knowledge of species distributions and potential zones of sympatry. Complimented with morphological and genetic data, such surveys would help resolve the status of subspecies and potentially identify undescribed species. Perhaps the greatest needs, however, lie in expanding our knowledge of the ecology of ice crawlers and the impacts of ongoing environmental change.
